# Correction: Divergent *Plasmodium* kinases drive MTOC, kinetochore, and axoneme organisation in male gametogenesis

**DOI:** 10.26508/lsa.202603692

**Published:** 2026-03-30

**Authors:** Ryuji Yanase, Mohammad Zeeshan, David JP Ferguson, Robert Markus, Declan Brady, Andrew R Bottrill, Anthony A Holder, David S Guttery, Rita Tewari

**Affiliations:** 1 School of Life Sciences, University of Nottingham , Nottingham, UK; 2 Department of Genetics, Genomics and Cancer Sciences, University of Leicester, Leicester, UK; 3 Nuffield Department of Clinical Laboratory Sciences and John Radcliffe Hospital, University of Oxford, Oxford, UK; 4 Department of Biological and Medical Sciences, Faculty of Health and Life Sciences, Oxford Brookes University, Oxford, UK; 5 School of Life Sciences, Gibbet Hill Campus, University of Warwick , Coventry, UK; 6 Malaria Parasitology Laboratory, Francis Crick Institute, London, UK

## Abstract

This study reveals how *Plasmodium* kinases regulate MTOC, axoneme and kinetochore organisation in male gametogenesis, providing key insights into potential targets for malaria transmission control.

Article: Yanase R, Zeeshan M, Ferguson DJP, Markus R, Brady D, Bottrill AR, Holder AA, Guttery DS, Tewari R (2025 March 24) Divergent *Plasmodium* kinases drive MTOC, kinetochore and axoneme organisation in male gametogenesis. Life Sci Alliance 8(6): e202403056. doi: https://doi.org/10.26508/lsa.202403056. PMID: 40127922.

Description of the correction:

In the originally published version of this article, an error was identified in Figure S2A. Specifically, incorrect images were inadvertently used for the top two differential interference contrast (DIC) panels.

**Figure S2.**
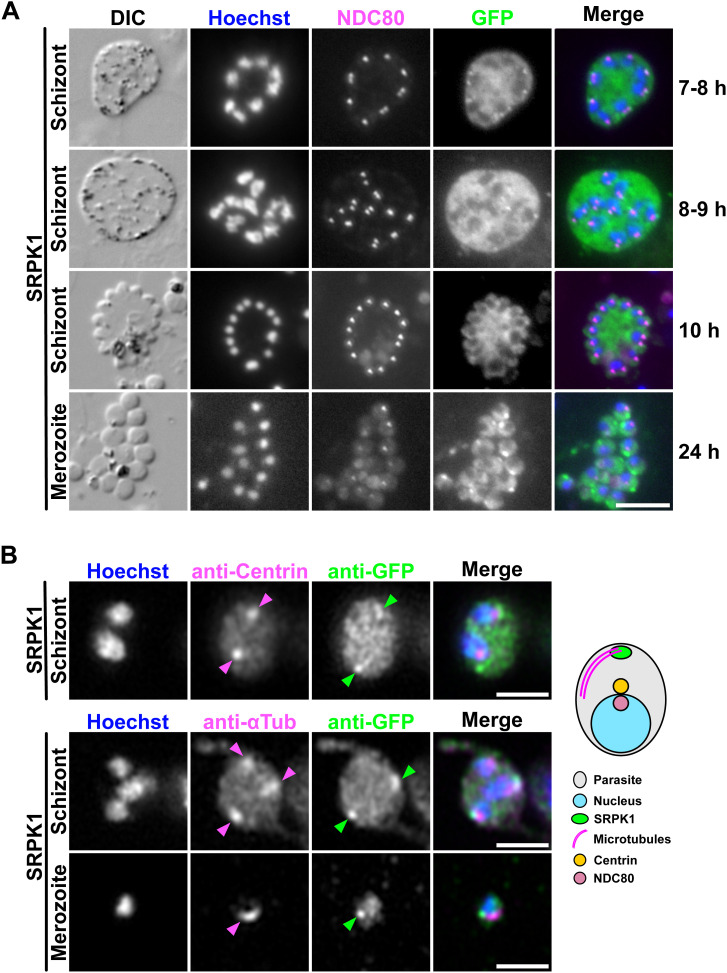
Co-localisation of SRPK1-GFP with kinetochore and centrin markers during asexual blood stages. **(A)** Localisation of GFP-tagged SRPK1 crossed with mCh-tagged NDC80 in blood-stage schizonts or merozoites. DIC, differential interference contrast. Merge shows Hoechst (DNA, blue), NDC80-mCh (magenta), and SRPK1-GFP (green). The time on the right indicates the duration within the schizont culture medium. Scale bar = 5 μm. **(B)** Immunofluorescence microscopy using a GFP-SRPK1 cell line with anti-GFP and anti-centrin or anti–α-tubulin antibodies. Merge shows Hoechst (DNA, blue), anti-centrin or anti–α-tubulin staining (magenta), and anti-GFP staining (green). Arrowheads show the fluorescence foci. Scale bars = 2 μm. The location of SRPK1 and each protein marker is summarised in the schematic diagram on the right.

To correct this error, we have replaced the incorrect panels with the appropriate DIC images. The newly revised and correct Figure S2 is provided (Fig S2).

Impact of this change:

We emphasise that this is a minor correction limited solely to the representative images shown in Figure S2A. This change does not affect the data analysis, the results described in the text, or any other figures. Therefore, it has no impact on the overall content, the interpretation of the data, or the scientific conclusions of the article.

We sincerely apologise to the readers and the editors for this oversight and any confusion it may have caused.

