# Supplementary material for: Divergent Plasmodium kinases drive MTOC, kinetochore and axoneme organisation in male gametogenesis
Source: Life Sci Alliance. 2025 Mar 24;8(6):e202403056. doi: 10.26508/lsa.202403056 (PMC11933671; doi:10.26508/lsa.202403056)
Supplement: Supplementary file 1 [file LSA-2024-03056_SdataFS1.pdf]

Source Data 1.

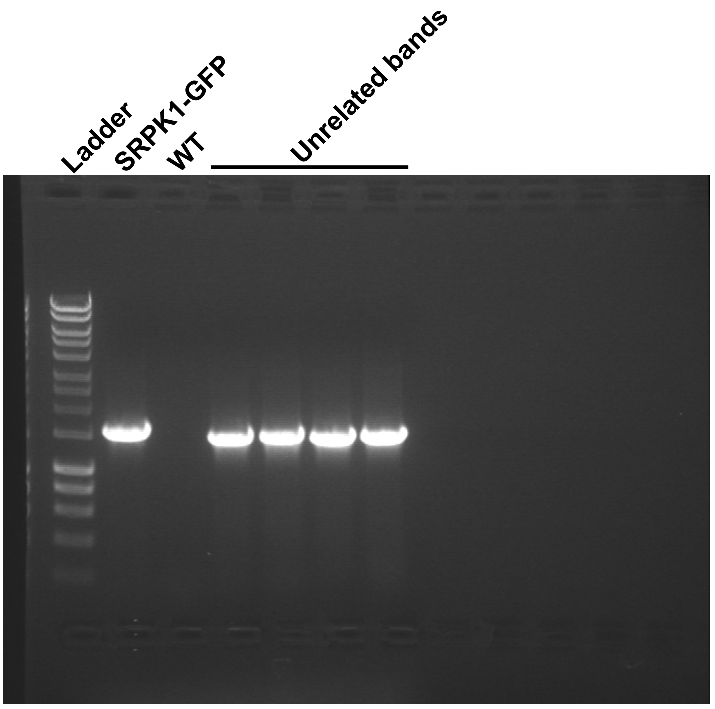

Figure S1B (SRPK1-GFP Integration PCR)

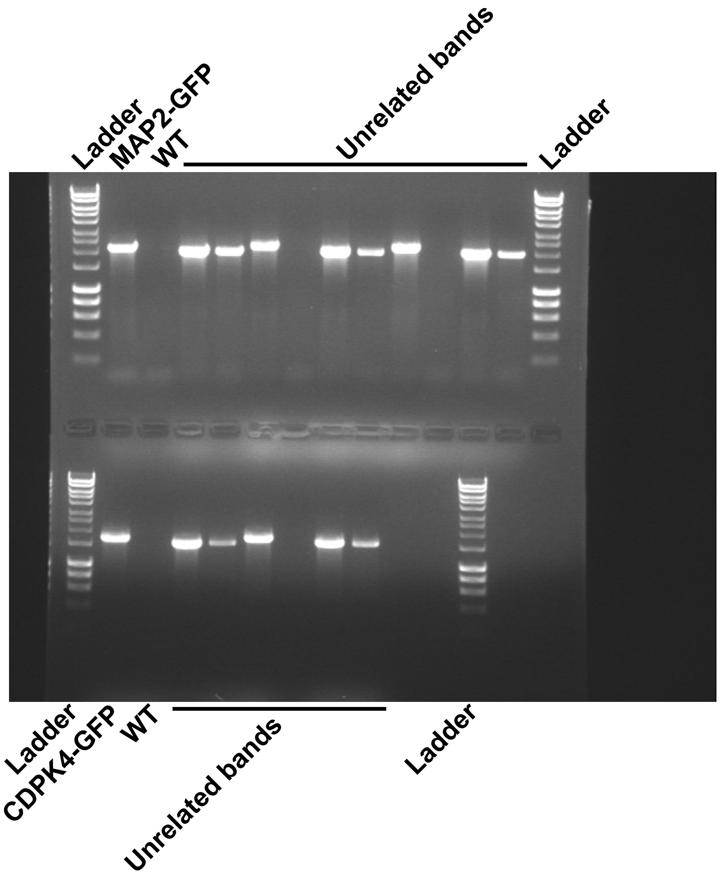

Figure S1B (MAP2-GFP and CDPK4-GFP Integration PCR)

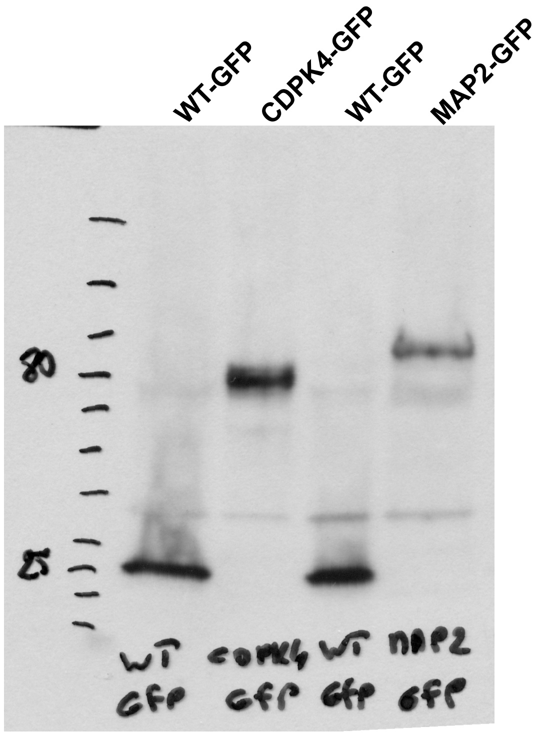

Figure S1B (CDPK4-GFP, MAP2-GFP Western blot)

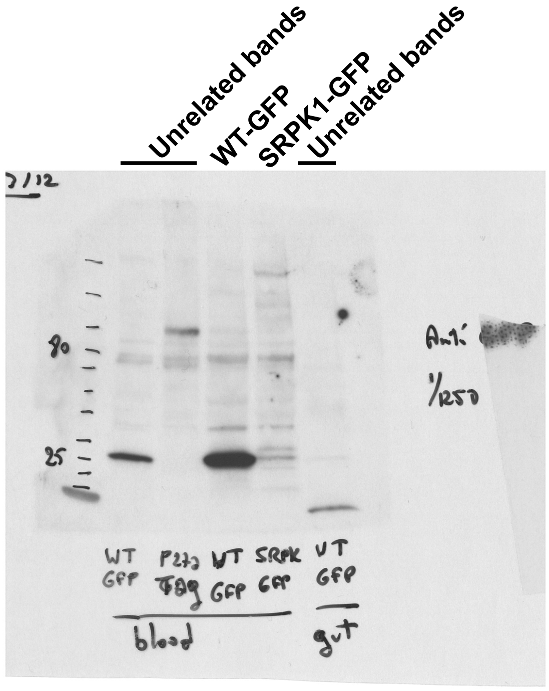

Figure S1B (SRPK1-GFP Western blot)
